# Exploring GVS as a display modality: cutaneous sensations and cue association maintenance

**DOI:** 10.1007/s00221-025-07058-z

**Published:** 2025-03-21

**Authors:** David R. Temple, Lanna N. Klausing, Brady C. Hogoboom, Abhishek Datta, Torin K. Clark

**Affiliations:** 1 University of Colorado Boulder (Smead Aerospace Engineering Sciences Department), Boulder, CO USA; 2 University of Colorado Anschutz Medical Campus (Department of Orthopedics), Aurora, CO USA; 3 Soterix Medical, Inc., Woodbridge, NJ USA

**Keywords:** Galvanic vestibular stimulation (GVS), Vestibular, Information transfer, Tactile, Cutaneous

## Abstract

Recent studies have investigated the potential use of Galvanic Vestibular Stimulation (GVS) as an alternative display modality. Such a GVS display could allow for parallel processing of information under increasing demands on other modalities (e.g., visual, auditory, or tactile), and perhaps be preferrable to other displays in certain circumstances (e.g., covert night operations). Prior studies quantified how precisely humans distinguish GVS cues modulated in the frequency, amplitude, or polarity of the sinusoidal burst of current, found cues to be robust to various environments, and have limited degradations in maintaining posture. Questions still arise though as to: (1) whether those receiving GVS cues respond primarily to vestibular or potentially cutaneous sensations, and (2) if multiple cues can be associated with different responses and if that capability can be maintained, which we addressed through two experiments. In the first, a topical anesthetic was not found to affect frequency and amplitude modulated GVS thresholds; however, polarity modulated GVS thresholds were elevated when cutaneous sensation was diminished. The second experiment revealed subjects distinguish among six different GVS cues composed of frequency (two conditions) and polarity (three conditions) modulations, and they maintained their association of these six cues three hours later. Collectively our results suggest that individuals are primarily responding to vestibular sensations when utilizing a GVS display and that quick association of at least six GVS cues to different responses can occur and be maintained at least three hours later. These findings continue to support the use of GVS as a viable display modality.

## Introduction

People obtain information about their environment through multiple senses, and the visual and auditory modalities are the most common means by which to display information to individuals. Oftentimes when a single display channel is overloaded with information, a “bottleneck” in human processing capabilities occurs where only a limited amount of information can be processed at once. However, often when multiple sensory modalities are utilized, the brain is capable of parallel processing, where it can integrate and analyze information from different senses at the same time (Wickens 1981, 2002, 2021; Meyer and Kieras 1997). This multisensory integration can allow for more efficient and effective information transfer, especially under demanding attentional tasks. For example, pilots are required to visually monitor a substantial number of instruments and displays during flight, an attentional task that is aided by auditory cues (e.g., various sounding alarms) if a system deviates from nominal.

Although common, visual and auditory display modalities are not the only means by which to relay information. Haptic displays, such as vibrating tactile vests, have been investigated as a means of improving navigation capabilities and situational awareness of soldiers in the field (Elliott et al. 2006, 2011, 2015; Hancock et al. 2015). Vestibular inputs (i.e., the inner ear organs that signal head motion), critical for self-orientation, balance, and stability, provide another potential modality with which to relay information. We have previously explored the potential to utilize the inner ear as a display modality through the use of Galvanic Vestibular Stimulation (GVS) (Smith et al. 2024; Temple et al. 2024) and have further tested its use presently.

There are certain scenarios where GVS may be preferential over other display types. For example, haptic displays could prove less effective in moving vehicles if users are unable to distinguish specific haptic cues from vehicle vibrations. Unlike certain tactile, auditory, or visual displays, vestibular stimulation can be provided without emitting any external sound or light or masking one’s field of view or hearing capabilities (e.g., attributes which could be beneficial for soldiers during covert, night operations). Furthermore, GVS devices have low power, mass, and volume requirements, making them easy to pack and utilize for extended durations. Despite the potential benefits of a GVS display modality, the idea is still rather new and warrants further investigation to determine effectiveness and understand the mechanisms by which GVS cues are sensed.

We previously found people are capable of distinguishing between cues of GVS “bursts” (i.e., 1 second in duration, 10–90 Hz sinusoidal current waveforms) varying in a number of current characteristics (frequency, amplitude, and polarity), under various conditions (e.g., seated, standing, walking, passively moving, under quiet or loud conditions), and in different environmental conditions (room temperature, hot, cold, and windy). Overall, we found individual performance on our thresholding tasks distinguishing between different GVS cues to be rather consistent and robust to the various testing conditions we imposed (Smith et al. 2024; Temple et al. 2024).

As GVS is known to impact postural control and generate postural reflexes, such as body sway towards the anode electrode (Coats and Stoltz 1969; Day et al. 1997; Séverac Cauquil et al. 2000; Fitzpatrick and Day 2004; St George and Fitzpatrick 2011), a concern for its use as a display modality is the potential for it to disorient or perturb balance during use. We have noted that sinusoidal, fast frequency (≥ 10 Hz), bipolar GVS cues (i.e., waveforms oscillating between current traveling in the left and right directions) did not disrupt postural control during a tandem walk task (Smith et al. 2024), which is consistent with prior research noting insignificant coherence between GVS currents and COP data beyond 3 Hz (Dakin et al. 2007). However, as muscles responsible for postural control are likewise known to exhibit significant coherence with GVS current frequencies of up to 20 Hz (Dakin et al. 2007), to limit GVS cue postural interference, it is likely advisable to utilize sinusoidal, bipolar current frequencies faster than 20 Hz. This argument can be further supported by recent research conducted by Wuehr, where subthreshold (i.e., amplitudes below sensory detection thresholds) noisy GVS frequencies ranging from 0 to 30 Hz (mostly within the range of < 20 Hz, that we would not advise for potential postural disruption at suprathreshold amplitudes) seemingly improved postural performance in the form of reduced center of pressure velocity (presumably via the stochastic resonance phenomenon). However, at higher amplitudes (e.g., 0.6 and 0.7 mA), several subjects appear to show masking effects, where these slower frequencies (i.e., < 20 Hz) at amplitudes likely detectable by the subjects (e.g., amplitudes one might choose to utilize as a GVS display) increased center of pressure velocity (Wuehr et al. 2024). Additionally, we previously found unipolar currents (i.e., those traveling in only one direction) to interfere with postural control, particularly in the absence of vision (Temple et al. 2024), consistent with numerous studies noting specific postural responses to GVS current directionality (Coats and Stoltz 1969; Day et al. 1997; Séverac Cauquil et al. 2000; Fitzpatrick and Day 2004; St George and Fitzpatrick 2011). Thus, if postural stability is of utmost importance for the task at hand, unipolar currents may be inadvisable. However, unipolar currents can produce rather salient directional cues associated with their postural responses (St George and Fitzpatrick 2011; Brunyé et al. 2024). Therefore, if relaying directional information is crucial (e.g., “turn left” or “turn right”), scenarios may exist where unipolar currents are appropriate (e.g., steering an individual towards a target). Ultimately, context is important when developing GVS cues and considering potential balance disruption, as is with any type of display. In fact, most modalities commonly utilized as displays also have the potential to induce body sway and disrupt postural control, including vision (Lee and Aronson 1974; Lee and Lishman 1975; Freeman et al. 2023), auditory sounds (Lubetzky et al. 2020; Jouira et al. 2025), and tactile belts (Lee et al. 2012, 2013). The vestibular system is another such modality likewise with that potential. Further research is warranted to develop GVS cues least likely to disrupt balance and interindividual differences should be considered.

Although it is well established that current amplitudes (e.g., 0.6 mA peaks) we have utilized previously (Smith et al. 2024; Temple et al. 2024) do modulate vestibular afferents (Goldberg et al. 1984; Abe et al. 2008; Kwan et al. 2019; Manca et al. 2019; Steinhardt and Fridman 2021; Forbes et al. 2023), as the current must likewise travel through the skin (Truong et al. 2024), cutaneous sensations can also be produced (e.g., prickling sensations) (Stone et al. 2025). Thus, it is still an open question as to whether individuals receiving GVS cues are responding primarily to vestibular sensations or potentially cutaneous sensations. Such a distinction matters if the goal of a GVS display is to relay information via a new modality (i.e., the vestibular system) as opposed to modalities which have been previously utilized (i.e., tactile/cutaneous inputs). For example, if those using a GVS display are primarily responding to cutaneous sensations, it is possible they may not see benefits of parallel processing capability when simultaneously utilizing a haptic display (e.g., a vibrating tactile vest). Although employing two separate displays, if both utilize the same modality, bottlenecking from limited processing capacity could still occur. This motivates research designed to probe the exact sensory modality that a GVS display utilizes.

To explore questions of parallel processing with a GVS display, we did investigate utilizing a dual-task paradigm (Temple et al. 2024). Subjects performed frequency and polarity thresholding tasks alone (baseline) and under conditions when a visual search task was concurrently performed. The visual search task did show some interference while performing the polarity thresholding task; however, the GVS thresholding tasks themselves were not impacted by dual-tasking. These findings provide some support of a GVS display’s potential use for expanding processing capabilities, though more research is warranted. Further, the exact modality (i.e., vestibular or tactile) that is primarily utilized with a GVS display should be explored.

Another area in need of further research involves the extent to which different GVS cues can be associated with various tasks and how well those associations are maintained (Temple et al. 2024). Our previous thresholding task investigations only required subjects to make a forced-choice distinction between two different types of stimuli at a time (i.e., frequency: faster or slower; amplitude: higher or lower; polarity: left or right) (Smith et al. 2024; Temple et al. 2024). GVS polarity cues have been found highly effective at relaying directions during a complex virtual navigation task, but the stimuli only relayed three specific directions (i.e., right, left, or straight) (Brunyé et al. 2024). As multiple aspects of GVS stimuli can be manipulated (e.g., frequencies, polarities, amplitudes, durations, etc.), there is the potential to relay a rich amount of information to individuals. How many various cues individuals can associate with specific responses deserves further investigation though.

Presently, we conducted two experiments to investigate GVS cue thresholding capabilities under conditions with diminished tactile sensitivity (Experiment 1) and investigate the capability of individuals to distinguish among and maintain associations of six different GVS cues (Experiment 2). In Experiment 1, we hypothesized that a topical anesthetic would significantly diminish tactile sensitivity compared to a placebo. Further, we hypothesized that GVS thresholding tasks (Frequency, Amplitude, and Polarity) would not be impacted by the topical anesthetic as compared to the placebo. For Experiment 2, we hypothesized that performance on a six, GVS cue association task would not differ three hours after an initial training session.

## Methods

Fourteen human test subjects between the ages of 19 and 32 years were recruited for the two experiments, with a total of 10 subjects in each experiment. As we did not have an estimated effect size for each of our hypotheses, a formal power analysis was not performed, but the sample size was defined prior to the start of data collected based upon other similar experiments (Smith et al. 2024; Temple et al. 2024). As was done in our previous study (Temple et al. 2024), inclusion criteria involved individuals that had no known paraben allergy (to mitigate allergic reaction risk from gels or creams), could stand and walk for several minutes and were not on any medication that could impact such abilities, had not recently consumed alcohol (within the last six hours), were not pregnant or breastfeeding, and had not fallen within the past year. Adherence to these criteria was self-reported beforehand with a questionnaire. Six subjects completed both experiments while four only completed Experiment 1 (4 M/6F) and another four only completed Experiment 2 (4 M/6F). Subjects were not permitted to participate in Experiment 2 on the same days as Experiment 1 sessions though. Experiment 1 measured thresholds at which subjects could distinguish between GVS stimuli with three parameter manipulations (i.e., Frequency, Amplitude, and Polarity), as we had done in a previous study (Temple et al. 2024). Subjects performed these thresholding tests two times each (i.e., first time = baseline, second time = post topical anesthetic/placebo), in one session where an over-the-counter, 5% lidocaine topical anesthetic cream (Numb Master, Clinical Resolution Laboratory, Inc, Brea CA, USA) was applied to the electrode sites to reduce cutaneous sensation, and another session where a placebo petroleum jelly (Vaseline^®^, Unilever, Englewood Cliffs, NJ, USA) was applied which would not reduce cutaneous sensations. Experiment 2 tested subject maintenance of six different GVS cue associations. Subjects first performed a training session, followed by a maintenance test session three hours later on the same day. GVS stimulators for these experiments were provided by Soterix Medical Inc. (Woodbridge, NJ, USA).

Informed consent was obtained for each subject prior to their enrollment in the study. All experiments described in this study were approved by the Institutional Review Board of the University of Colorado Boulder (protocol #22–0110), complying fully with the tenets of the Declaration of Helsinki.

### Procedure

All testing was performed with the subjects seated in a chair. We began subject preparations for each experiment by disinfecting the skin behind the ears (over the mastoid processes) using alcohol wipes. This area was then exfoliated using NuPrep Skin Prep Gel (Weaver and Company, Aurora, CO, USA). Alcohol wipes were used once more to remove remaining residue from the area. Study staff then proceeded with experiment-specific preparation.

### Experiment 1

**Fig. 1:**
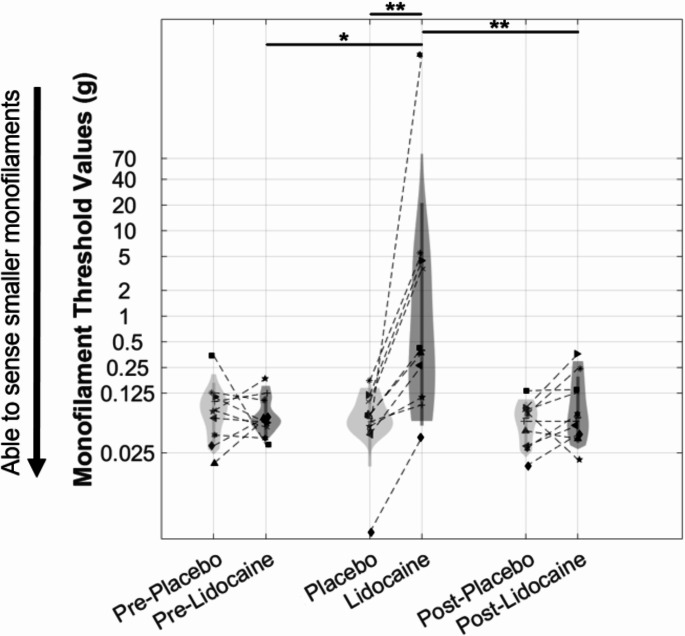
Violin plots representing subject distributions of the monofilament tactile thresholds. Each subject is indicated with a symbol and connected by dashed lines. The vertical axis is natural log scaled in order to help better distinguish individual data. Vertical rectangular boxes indicate the ± 1 standard deviation range. Asterisks denote significant comparisons without (*p < 0.05) and with (**p < 0.01) Bonferroni adjustments for multiple comparisons

For Experiment 1, subjects participated in two sessions, separated by 22–48 h. At the start of each visit, test administrators performed a series of baseline monofilament tests on the subject’s freshly cleaned mastoid processes (referred to as Pre-Lidocaine or Pre-Placebo). These tests were modified from standard procedures utilized for foot and hand tactile sensitivity tests (Bell-Krotoski and Tomancik 1987; Holewski et al. 1988; Bell-Krotoski et al. 1995). This consisted of applying five presses to the mastoids using monofilaments of 4 g, 2 g, 0.4 g, 0.07 g, 0.04 g, and 0.02 g each, in decreasing order of weights. Subjects were instructed to verbally report each press they felt immediately as they perceived it on their skin, while study staff recorded the number of correctly detected perceptions out of five. Subjects were not informed of their performance during these monofilament tests. If subjects hesitated or did not respond immediately after a press, this was considered a miss. This was performed on both the left and right mastoids at each monofilament weight. From the proportion of correctly detected monofilament tests, as a function of monofilament weight (on a log-transformed axis), we fit a cumulative Gaussian psychometric curve. The µ value of the cumulative Gaussian corresponds to 50% likelihood of correctly detecting the monofilament press, which we took as the tactile sensitivity threshold, reported in units of monofilament weight. A threshold was computed for each subject, in each condition (placebo and lidocaine), at each time point (before application or Pre-Lidocaine/Pre-Placebo, immediately after application or Lidocaine/Placebo, and after GVS testing or Post-Lidocaine/Post-Placebo, in Fig. 1), with data pooled across the left and right mastoid following visualization that there were not any noticeable differences.

After completing this baseline tactile measurement (Pre-Lidocaine or Pre-Placebo), study staff then prepared the mastoids with either the topical anesthetic (i.e. 5% lidocaine cream) or placebo (i.e., Vaseline^®^). The order of application was counterbalanced between the two visits. Subjects were blinded to which cream they received on either day. A cotton bud was used to liberally (<½ tsp per mastoid) apply the selected cream to the area in which the electrodes were to be placed. The cream was rubbed into the skin for approximately 30s, and then a clear bandage was placed to cover the entire area. The subjects were fitted for the electrode headband and plastic gel cups, and a 30-minute timer was set to allow for the cream to activate. This timer was utilized whether it was the placebo or the lidocaine conditions. At the end of the 30 min, the headband and bandages were removed, and the cream was cleaned from the skin using alcohol wipes. A stopwatch was set after the cream removal, to keep track of time since the lidocaine/placebo had been removed.

At this point, another round of monofilament testing was recorded on each mastoid (called Lidocaine or Placebo, depending upon which had been applied), following the same procedure detailed above to quantify any reduced cutaneous sensation. Upon completion of the monofilament test, the already-fitted GVS headband was donned, with cups filled with electrolyte gel (HD-GEL, Soterix Medical Inc., Woodbridge, NJ, USA) placed over the mastoids holding the electrodes. Impedance was checked and ranged from 0.6 to 11 kΩ across subjects.

Subjects then performed a series of three GVS thresholding tests; the order was counterbalanced between subjects but was repeated between session day one and day two. These were referred to as the Frequency, Polarity, and Amplitude thresholding tests. Methods for these tests were the same as detailed in our previously published works (Smith et al. 2024; Temple et al. 2024). Subjects performed 50 trials for each (Frequency, Polarity, and Amplitude) thresholding test, after first being given a few example stimuli with feedback to familiarize subjects to each of the tests and confirm they understood the task.

Frequency thresholding aimed to measure the just-noticeable difference (JND) frequency threshold value which subjects could reliably differentiate between currents faster or slower than 50 Hz. All cues were bipolar sinusoidal waves +/-0.6 mA, one second in duration. Subjects were given a pedestal “Stimulus A” of 50 Hz, followed by a “Stimulus B” of 50 +/-40 Hz, and subjects indicated if Stimulus B felt “faster” or “slower” than the pedestal (Stimulus A). This two-alternative forced-choice (2AFC) test utilized a three-down, one-up (3D1U) staircase algorithm requiring three correct responses in a row before narrowing the stimuli window (a Δ closer to the 50 Hz pedestal), and one incorrect response increased (a Δ further from the 50 Hz pedestal) the stimuli window.

The Amplitude thresholding test aimed to measure the JND amplitude threshold value which a subject could reliably distinguish between currents stronger or weaker than 0.6 mA. All Amplitude thresholding cues were bipolar, sinusoidal waves at 50 Hz, and one second in duration. Subjects were given a pedestal Stimulus A of 0.6 mA, followed by a Stimulus B of 0.6 mA +/- 0.5 mA, and indicated if Stimulus B was “stronger” or “weaker” than the pedestal (Stimulus A). Amplitude thresholding similarly used a 2AFC test with a 3D1U algorithm to narrow (a Δ closer to the 0.6 mA pedestal) or widen (a Δ further from the 0.6 mA pedestal) the amplitude stimuli window of Stimulus B.

The Polarity thresholding test aimed to measure the lowest peak current amplitude that subjects could reliably differentiate the localized direction of current flow (i.e., from electrode on left mastoid to right or vice versa). Subjects were presented with a single, one second duration stimulus of 50 Hz, varying in amplitude and polarity (current either traveling to the left or right). The 3D1U staircase adjusted the stimulus amplitude (beginning at 1 mA), while polarity was randomized for each trial. Subjects reported if they felt the stimulus on the “left” or “right” side, and a direction recognition threshold computed the Polarity threshold as the peak current amplitude subjects could reliably distinguish between left and right polarity cues. Stimuli delivered to subjects in this test were capped at a maximum amplitude of 1 mA, and could be delivered as small as 0.1 mA.

At the end of the three thresholding tests, the GVS headband was removed, and any remaining gel was wiped away using alcohol wipes and paper towels. A final monofilament test was performed (called Post-Lidocaine or Post-Placebo in Fig. 1), and the time from the initial removal of the cream was noted. The time from removal of the placebo/lidocaine cream to completion of the final monofilament test ranged from 35 to 108 min (median = 54); however, only two of the subjects completed GVS thresholding tests during the lidocaine session that took longer than the product’s claimed effective numbing time of one hour.

### Experiment 2

Experiment 2 took place across two sessions, separated by three hours, with each session lasting approximately one hour. At the start of the first session, the subjects’ skin was prepared as detailed above and the electrolyte gel was again used to fill the electrode cups positioned on the mastoid processes. Subjects were then introduced to the components of the GVS cues they would be receiving during the experiment. The GVS cues were one second in duration, 0.8 mA in amplitude, and consisted of a frequency (either a fast, 65 Hz or slow, 35 Hz sinusoidal frequency) and polarity component (unipolar left, unipolar right, or bipolar), yielding a total of six different GVS cues (two frequencies × three polarities) to associate with specific responses. Each component (e.g., slow, fast, left, right, center/bipolar) was introduced to the subject first, then training on how to report each of the six potential cues was performed. A graphical user interface (GUI) developed in MATLAB (MathWorks, Natick, MA, USA) displaying a button for each of the six cue choices (i.e., fast left, fast center, fast right, slow left, slow center, or slow right) was utilized to record subject responses, as well as provide subjects feedback during the training. The GUI response button turned green when the correct response was given, and if an incorrect response was chosen, the answer that should have been chosen turned red. Subjects were instructed how to utilize the GUI, then 48 training trials were performed, with each of the six possible GVS cues being presented eight times in a randomized order.

After training, an initial cuing test was performed with another 48 GVS cue trials (each of the six GVS cues again presented eight times in a different randomized order) utilizing the GUI, but with no feedback provided (buttons did not change colors to indicate correct or incorrect responses). Once the initial cuing test was complete (Session 1), the electrodes were removed, and any remaining gel was wiped away. Subjects were then free to leave for three hours before coming back for the second session.

When subjects arrived back on the same day for the second session, they were again prepped behind the ears for electrode placement. Alcohol wipes were used once more to clean the mastoid processes, and the electrode cups holding the electrodes were again filled with the electrolyte gel and placed over the mastoids. NuPrep Skin Prep Gel was not used to prep subject skin on the return session, as it was only three hours later, and we did not want to irritate the skin from too much exfoliation. A second, maintenance cuing test (Session 2) was then performed with 48 GVS cue trials (each of the six GVS cues presented eight times in another randomized order) utilizing the GUI, again with no feedback provided. Once the Session 2 GVS cuing test was complete, the electrodes were removed, any remaining gel was wiped away, and the subjects were free to leave.

### Statistical analysis

For monofilament tactile thresholds in Experiment 1, we found (see Fig. 1) that a few subjects had very low or very high tactile thresholds that exceeded the ranges of the monofilament weights we tested. These were in the direction that we would have expected; specifically, one subject immediately after having lidocaine applied had a tactile threshold computed as 1153 g (well above the maximum monofilament weight of 4 g, corresponding to not being able sense any of the monofilaments), and another subject in a placebo condition had a computed tactile threshold of 0.003 g (below the minimum monofilament weight of 0.02 g, corresponding to being able to sense all monofilament presentations). While these tactile thresholds should be interpreted as reliably being very large and very small, respectively, because there were no monofilament presentations above (and below, respectively) to bound the threshold, we did not employ statistical analysis which relied upon the exact computed threshold. Instead, we used a non-parametric test, which simply assessed if the tactile thresholds were higher or lower within subjects across the conditions/time points (before application or Pre-Lidocaine/Pre-Placebo, immediately after application or Lidocaine/Placebo, and after GVS testing or Post-Lidocaine/Post-Placebo). Specifically, we used a Friedman test (equivalent to a one-way repeated measures ANOVA) with six levels (Pre-Placebo, Placebo, Post-Placebo, Pre-Lidocaine, Lidocaine, Post-Lidocaine). This was followed by non-parametric Wilcoxon signed rank tests between a subset of the relevant pairings: each lidocaine test and the corresponding placebo test, and between lidocaine tests at different timepoints (pre vs. during, and during vs. post), reported prior to and with Bonferroni corrections for these five relevant comparisons. Reporting of both Bonferroni adjusted and unadjusted corrections was done for transparency, such that readers may distinguish whether the lack of significance (e.g., Pre-Placebo vs. Pre-Lidocaine) was due to applying a highly conservative Bonferroni multiple-comparison correction or would have been non-significant even without any correction.

For the GVS thresholding tests in Experiment 1, significant Shapiro-Wilk tests (*p* < 0.05) indicated that the GVS thresholds (Frequency, Amplitude, and Polarity) from Experiment 1 were not normally distributed. Therefore, a natural log transformation was applied to the GVS thresholding data, which allowed the Frequency and Amplitude thresholds to meet the assumption of normality (Shapiro-Wilk tests; *p* > 0.05). Two-tailed, paired *t*-tests were then used to assess significant differences between the Lidocaine and Placebo sessions for transformed Frequency and Amplitude GVS thresholds. For Polarity thresholds, log transformation still did not conform to a normal distribution (Shapiro-Wilk test; *p* = 0.002), so a nonparametric Wilcoxon signed rank test was utilized to assess significant differences between the Lidocaine and Placebo sessions for Polarity thresholds. As we assessed only one comparison (Lidocaine vs. Placebo) for the three separate dependent variable metrics (Frequency, Amplitude, and Polarity thresholds), *p*-value corrections for multiple comparisons were not performed. Further, any correction would have made significant comparisons less likely to occur and increase the likelihood of confirming our expectations. Thus, to be transparent and avoid reporting insignificant findings potentially due to *p*-value corrections, such corrections were not applied.

**Fig. 2:**
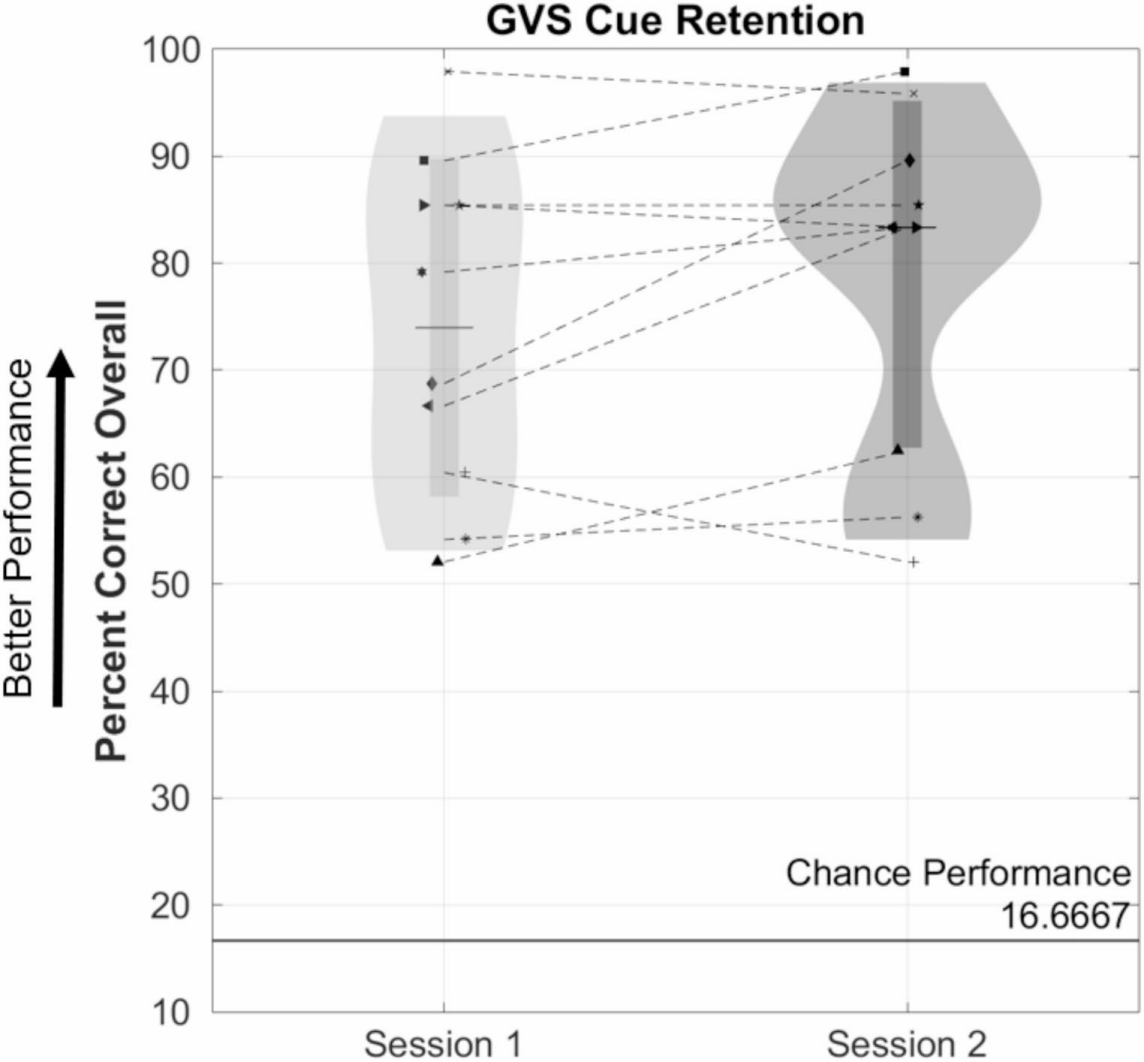
Violin plots representing subject distributions of correct response percentages during tests of the six GVS cues. Symbols are the same as in Fig. 1. No significant differences were noted between Session 1 and Session 2 (performed three hours later). The long bolded horizontal line at the bottom represents the percentage of cues subjects would have been expected to get correct by chance (16.67%)

For Experiment 2, correct response percentages were calculated for each subject during the Session 1 cue identification test and the three-hour-later Session 2 maintenance test. A two-tailed, paired *t*-test compared differences between Sessions 1 and 2 to determine if there was any significant loss of association among the six GVS cues and their corresponding responses. In addition to comparing whether subjects’ full response was correct (Percentage Correct Overall, Fig. 2), we also performed analyses comparing whether subjects got part of the response correct (either the frequency or polarity aspect of a cue). As with the GVS thresholding data from Experiment 1, we did not expect significant differences in percentage correct between Session 1 and Session 2. Thus, *p*-value corrections were not performed, to avoid reporting insignificant findings potentially due to such corrections.

## Results

### Experiment 1: quantifying thresholds with Lidocaine

As seen in Fig. 1, the monofilament tactile thresholds were dramatically increased after having applied the Lidocaine, as expected. A non-parametric Friedman test of differences among the 6 repeated measures presentations (Pre-Placebo, Placebo, Post-Placebo, Pre-Lidocaine, Lidocaine, Post-Lidocaine) was conducted and revealed significant differences across conditions (χ²(5) = 18.46, *p* = 0.0024). Non-parametric Wilcoxon signed rank tests found significantly elevated monofilament tactile thresholds following Lidocaine application (*Mdn* = 0.4, *n* = 10) vs. Placebo application (*Mdn* = 0.06, *n* = 10), *W + =* 0, *p* = 0.002, *p*_*adj*_ = 0.01 (with Bonferroni for five follow-up comparisons reported on monofilaments), *r* = -1, as expected. The effect was similar following Lidocaine application (*Mdn* = 0.4, *n* = 10) compared to Pre-Lidocaine (*Mdn* = 0.06, *n* = 10), *W + =* 6, *p* = 0.027, *p*_*adj*_ = 0.14, *r* = -0.7. Thus, the application of lidocaine increased the tactile threshold by over 6x. However, the Post-Lidocaine (*Mdn* = 0.07, *n* = 10) tactile threshold (performed following the GVS threshold testing, a median of 54 min after lidocaine was removed) was significantly lower than immediately following Lidocaine application (*Mdn* = 0.4, *n* = 10), *W + =* 54, *p* = 0.004, *p*_*adj*_ = 0.02, *r* = 0.9, and not different than Pre-Lidocaine (*Mdn* = 0.06, *n* = 10), *W + =* 20, *p* = 0.49, *p*_*adj*_ = 1, *r* = -0.2, or the Post-Placebo threshold (*Mdn* = 0.06, *n* = 10), *W + =* 11, *p* = 0.11, *p*_*adj*_ = 0.53, *r* = -0.5. This suggests that while lidocaine effectively increased tactile thresholds, the effect did not last throughout the testing session, returning to the baseline value (i.e., Pre-Placebo or Pre-Lidocaine). As expected, there were no differences between tactile thresholds from before, during, or after placebo application.

**Fig. 3:**
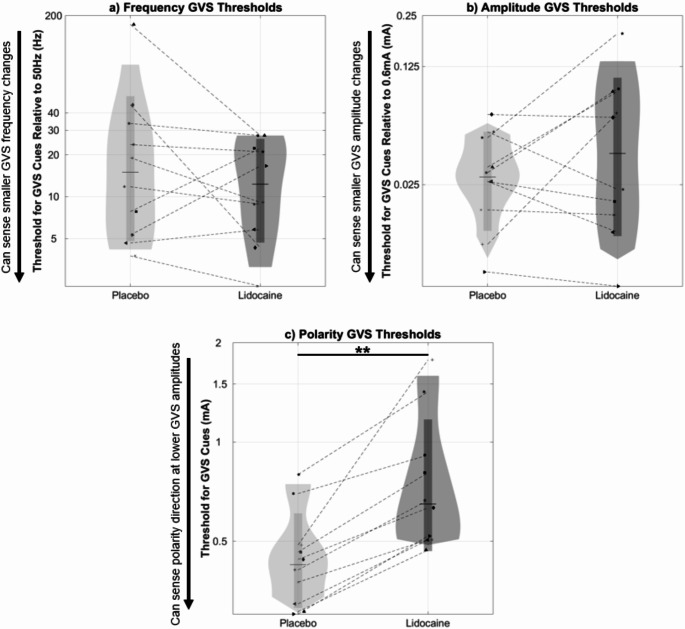
Violin plots representing subject distributions of (**a**) Frequency, (**b**) Amplitude, and (**c**) Polarity GVS thresholds, following Placebo or Lidocaine applications. Vertical axes are natural log scaled, and symbols are the same as in Fig. 1. Horizontal lines represent the median of GVS thresholds. No significant differences in Frequency or Amplitude GVS thresholds were noted between the Placebo and Lidocaine sessions, but asterisks denote significantly larger Polarity GVS thresholds in sessions when Lidocaine was applied, compared to the Placebo (**p < 0.01)

In terms of distinguishing between GVS cues, paired *t*-tests did not indicate any significant differences between the Lidocaine (*M* = 2.4, *SD* = 0.9) and Placebo (*M* = 2.8, *SD* = 1.2) sessions for the transformed Frequency thresholds, *t*(9) = 1.0, *p* = 0.33 (Fig. 3a). Transformed Amplitude thresholds likewise did not indicate significant differences between the Lidocaine (*M* = -3.3, *SD* = 1.1) and Placebo sessions (*M* = -3.6, *SD* = 0.7), *t*(9) = 1.1, *p* = 0.28 (Fig. 3b). These findings indicate subjects generally did not perform worse on these GVS thresholding tasks, despite reduced tactile sensation. However, Polarity GVS thresholds were significantly higher during the Lidocaine session (*Mdn* = 0.6, *n* = 10) compared to the Placebo session (*Mdn* = 0.4, *n* = 10), *W + =* 0, *p* = 0.002, *r* = -1, via a Wilcoxon signed rank test (Fig. 3c), indicating these thresholds were elevated when tactile sensation was diminished.

### Experiment 2: GVS cue-response association maintenance

Figure 2 shows performance in identifying 6 GVS cues (modulated in sinusoidal frequency at either 35–65 Hz and polarity (unipolar left, unipolar right, or bipolar), following a brief training (Session 1) and then after three hours and a new electrode application (Session 2). A paired *t*-test did not indicate any significant differences between Session 1 (*M* = 74, *SD* = 15.8) and Session 2 (*M* = 79, *SD* = 16.2), *t*(9) = 1.7, *p* = 0.12, with all subjects performing much better than would be expected by chance during both sessions. In fact, median percentages were slightly better in Session 2 than Session 1. These findings indicate a strong maintenance of GVS cue associations after three hours, and the ability of all participants to reliably distinguish among the six GVS cues.

In addition to considering the Percentage Correct Overall (i.e., correctly identifying both the frequency and polarity components of a cue), we also computed (not shown graphically) the percentage of correct responses for just frequency (independent of whether the polarity component was correctly identified or not) and the percentage of correct responses for just polarity (ignoring whether frequency was properly identified as fast vs. slow). Neither the percentage of correct frequency nor polarity responses showed a significant difference between Session 1 and Session 2. Furthermore, in each case, every subject performed above chance (median percentage correct across subjects and sessions for frequency = 84.4%, compared to 50% by chance; median for polarity = 97.9%, compared to 33% by chance), showing a strong maintenance of GVS cue response associations, for both frequency and polarity manipulations.

## Dicussion

### Experiment 1: using Lidocaine to investigate sensing modalities of GVS cues

In our first experiment, we investigated whether GVS display cues were predominantly sensed by the vestibular system or potentially by cutaneous tactile cues, by manipulating tactile sensitivity through the application of a topical anesthetic (Lidocaine) to the electrode site. We found GVS Frequency and Amplitude thresholds were not significantly different under diminished cutaneous sensation (after Lidocaine application, as compared to Placebo). This suggests distinguishing Frequency and Amplitude GVS cues are predominantly done via vestibular sensing.

On the other hand, GVS Polarity thresholds were significantly elevated following Lidocaine application compared to Placebo. This suggests that GVS cues utilizing Polarity manipulations (unipolar leftward vs. unipolar rightward waveforms) are distinguished, at least partially, using tactile cutaneous cues. While all 10 subjects had higher Polarity GVS thresholds with Lidocaine vs. Placebo, we note that the increase was relatively slight (median of 0.42 mA with Placebo vs. 0.65 mA with Lidocaine corresponding to an increase of a 55%). This is in contrast with the 6x increase in monofilament tactile threshold immediately following Lidocaine application (median of 0.064 g with Placebo to 0.40 g with Lidocaine). While the tactile threshold and GVS Polarity threshold should not be formally compared (different units and definitions of “threshold”), the dramatic reduction in tactile sensitivity from Lidocaine application yielding only a correspondingly slight increase in Polarity threshold suggests even GVS Polarity cues are sensed more so by the vestibular system than through tactile stimulation.

In general, our results for Frequency, Amplitude, and Polarity GVS thresholds suggest that distinguishing differences in GVS cues is largely done via vestibular sensing, rather than tactile cutaneous sensations. While this has previously been suggested (Brunyé et al. 2024), to our knowledge, this is the first systematic assessment of GVS thresholds when manipulating cutaneous tactile sensitivity. Our finding that GVS cue distinction primarily involves vestibular sensing, suggests that such a display could allow for parallel processing of information if utilized in combination with other cutaneous/tactile displays, such as tactile belts or vests (Elliott et al. 2006, 2011, 2015; Hancock et al. 2015). Future research should be done to confirm this suggestion though and determine with what other types of displays GVS is effective at reducing bottlenecking and improving information transfer.

### Experiment 2: association maintenance of multiple GVS cues

Previously, literature has quantified the precision in distinguishing GVS cues when presented in two, sequential intervals, differing in frequency (average of just noticeable difference of +/- 12 Hz relatively to a pedestal of 50 Hz) (Smith et al. 2024) or amplitude (median just noticeable difference of +/-0.03 mA relative to a pedestal of 0.6 mA) (Temple et al. 2024). Further, identifying the direction (left vs. right) unipolar currents travel has also been quantified at a median amplitude of 0.55 mA (Temple et al. 2024). However, all of these forced-choice tasks involved only two alternatives and a single manipulation. For GVS displays to encode rich information, it is important to assess whether several cues can be associated with particular responses, following a brief training session with feedback.

Presently, we found that six cues (each a combination of frequency: 35–65 Hz and polarity: unipolar left, unipolar right, or bipolar) could be reliably distinguished and associated following a brief training (roughly 10 min). While all 10 subjects performed well above chance (median of 74% in Session 1, compared to chance with six options being 16.67%), some individuals were much better at properly associating cues (ranging from 52 to 98% correct out of 48 presentations). This may suggest that our standardized presentation of GVS cues (1 s in duration, 0.8 mA, for either 35 Hz vs. 65 Hz, and unipolar left vs. unipolar right vs. bipolar) was insufficient for some subjects to reliably perceive.

We selected the 0.8 mA as the standard current amplitude for Experiment 2 based upon prior quantification of the median amplitude threshold of 0.55 mA for distinguishing between left and right unipolar currents (Temple et al. 2024). However, in this separate cohort of subjects, these Polarity thresholds ranged from 0.32 to 0.83 mA, so 0.8 mA may have been an insufficient current amplitude for some individuals to distinguish among the current polarity options in Experiment 2. Further, distinguishing between bipolar, as well as left and right unipolar may have been more challenging. Similarly, we selected 35 Hz vs. 65 Hz frequencies based upon just notice difference thresholds ranging from 5.4 to 19.6 Hz (median = +/-12 Hz) relative to a pedestal of 50 Hz, though this used exclusively bipolar waveforms (Smith et al. 2024), rather than the unipolar also presented here. Future work should consider higher amplitudes (and/or larger frequency differences) if presenting standard GVS cues or personalizing the amplitude or frequency difference to each individual’s sensitivity when encoding information. This is feasible with GVS cues, such as in Brunyé et al. (2024) where GVS amplitudes were customized to each individual when providing directional guidance associations (left, right, or straight). It is also possible that performance could improve with additional practice or longer training durations, which could likewise be explored in future studies.

Remarkably, following three hours (and the reapplication of electrodes) in a second session, correct associations of the six cues were maintained, with no difference in performance from the first session. This indicates that associations of the GVS cues to the desired responses were maintained well. In fact, there was a slight increase in the median percentage correct from 74% in Session 1 up to 83% in Session 2, which suggests associations were further engrained (although differences were not significant between sessions). Critically, the removal and reapplication of GVS electrodes between sessions, without any retraining with the second application suggests that a GVS display can be robust to miscellaneous variations in electrode placement and application.

### Limitations, considerations, and future work

Regarding Experiment 1, it is important to note that the anesthetic’s effectiveness at numbing cutaneous sensation did diminish with time, and two subjects continued with GVS threshold tests past the expected efficacy of the lidocaine (i.e., past one hour). However, the majority (8/10 subjects) did perform all GVS thresholds within the manufacturer’s suggested one-hour effectiveness period, thus we believe the majority of GVS thresholds were performed with diminished cutaneous sensation. Lidocaine works as a voltage-gated sodium channel inhibitor (Wehrfritz et al. 2011; Derry and Moore 2014), with the potential to nonselectively impede all primary cutaneous sensory receptor types (i.e., tactile corpuscles, mechanoreceptors, nociceptors, and thermoreceptors) and their afferent pathways within the skin. However, as vestibular induced afferents originate from the inner ear, within the temporal bone of the skull, we would not expect any such sensation loss caused by the lidocaine. Although our over-the-counter lidocaine cream was unable to ensure complete cutaneous sensation loss (i.e., subjects being unable to feel any monofilaments with lidocaine) such that GVS would be unable to stimulate any type of cutaneous sensation, we assumed if subjects primarily relied on cutaneous sensations to distinguish between cues that the GVS thresholds would have been increased by the diminished cutaneous sensation induced by the lidocaine, which was not the case for Frequency and Amplitude tests.

We note that one participant had an extremely high GVS Frequency threshold with Placebo (Fig. 3a). At 173 Hz for a just noticeable difference threshold relative to the 50 Hz pedestal, this is not representative of any other subjects tested in this study and others (Smith et al. 2024; Temple et al. 2024), and it is likely due to extraneous factors (misunderstanding the task, fatigue, boredom, apathy, etc.). However, their threshold with Lidocaine was more typical of other subjects. With the log transformation, this elevated threshold was not considered an outlier and was conservatively included in data analysis. Additionally, repeating our statistical analysis with this subject excluded did not change the conclusion that GVS Frequency thresholds were similar with Lidocaine as with Placebo.

It is also worth noting that although we made attempts to “blind” subjects as to whether they were receiving the Lidocaine or Placebo in Experiment 1, as the Lidocaine has a numbing effect that we then tested for with monofilaments, it was not possible with our design to truly “blind” subjects as to whether they received the Lidocaine or Placebo. With subjects in which the numbing effect was particularly effective, it is likely that they could have noticed more difficulty perceiving monofilaments and been aware of which they received. We do believe though that any effects of the Lidocaine on the tested GVS thresholds were primarily induced by the intended numbing effect the cream produced, rather than any extraneous variable.

Regarding Experiment 2, while this initial evaluation demonstrated the feasibility of rapidly associating six unique GVS cues to specific responses, future work should explore the limits of conveying richer information by encoding additional cues. We originally attempted pilot testing with twelve cues (two frequencies x three polarities x two amplitudes e.g., with a low: 0.8 vs. high: 1 mA amplitude), but found that stimuli ≥ 1 mA could be uncomfortable for some pilot subjects. Furthermore, during lower amplitude stimuli (≤ 0.8 mA), pilot subjects had more difficulty reliably distinguishing between the slow (35 Hz) vs. fast (65 Hz) frequencies. These findings caused us to reduce our present study design to six unique GVS cues (at one amplitude of 0.8 mA) for our full Experiment 2, but future work should continue to explore variations in GVS cue encoding, as more unique associations are likely feasible. Likewise, we found excellent cue association maintenance at up to three hours; however, future work could also investigate extended periods of time.

Finally, although we have evaluated short, GVS “bursts” presently, just as prior research has done (Smith et al. 2024; Temple et al. 2024), this is only one means of conveying information via GVS, and there are many other options that could be explored. As GVS has the potential to disorient and perturb capabilities such as postural control and locomotion (Bent et al. 2000; MacDougall et al. 2006; Moore et al. 2006; St George and Fitzpatrick 2011) though, the design of cues for such a display modality is important. As we alluded to in the introduction, there may be a tradeoff in some reduced postural stability when utilizing binaural, unipolar currents (Temple et al. 2024) or slower frequency bipolar currents below 20 Hz that fall within the range of typical vestibular evoked postural reflexes (Dakin et al. 2007). However, the typical postural responses induced by such GVS currents with those characteristics can also induce salient directional cues (St George and Fitzpatrick 2011), which may be appropriate for some navigational tasks (Brunyé et al. 2024). Ultimately though, the tasks being performed and how specific current aspects can affect those tasks must be considered when designing cues for a GVS display.

## Conclusions

Our present findings continue to support GVS as a possible display modality with low power, mass, and volume requirements, that can provide an effective way to relay information. We provide evidence that suggests those distinguishing among various GVS cues are primarily responding to vestibular, rather than cutaneous sensations. This finding infers that a new modality (i.e., the vestibular system) is being utilized in the processing of the cues and thus lends support to parallel processing capabilities with a GVS display, which could reduce bottleneck occurrence under demanding attentional tasks via multisensory integration. Furthermore, our study indicates people can quickly distinguish among six separate GVS cues and maintain that capability for at least three hours. Together this foundational work helps to pave the way for more operational implementation studies (Brunyé et al. 2024), which are needed to employ viable GVS displays.

## Data Availability

Data from this research will be made available upon reasonable request.

## References

[CR1] Abe C, Tanaka K, Awazu C, Morita H (2008) Strong galvanic vestibular stimulation obscures arterial pressure response to gravitational change in conscious rats. J Appl Physiol 104:34–40. 10.1152/japplphysiol.00454.2007 17916676 10.1152/japplphysiol.00454.2007

[CR2] Bell-Krotoski J, Tomancik E (1987) The repeatability of testing with Semmes-Weinstein monofilaments. J Hand Surg Am 12:155–161. 10.1016/S0363-5023(87)80189-2 3805636 10.1016/s0363-5023(87)80189-2

[CR3] Bell-Krotoski JA, Fess EE, Hiltz D (1995) Threshold detection and Semmes-Weinstein monofilaments. J Hand Ther 8:155–162. 10.1016/s0894-1130(12)80314-0 7550627 10.1016/s0894-1130(12)80314-0

[CR4] Bent LR, McFadyen BJ, Merkley VF et al (2000) Magnitude effects of galvanic vestibular stimulation on the trajectory of human gait. Neurosci Lett 279:157–160 10688053 10.1016/s0304-3940(99)00989-1

[CR5] Brunyé TT, Navarro E, Hart-Pomerantz H et al (2024) Guiding human navigation with noninvasive vestibular stimulation and evoked mediolateral sway. J Cogn Enhanc 8:54–64

[CR6] Coats AC, Stoltz MS (1969) The recorded body-sway response to galvanic stimulation of the labyrinth: A preliminary study. Laryngoscope 79:85–103 4303843 10.1288/00005537-196901000-00004

[CR7] Dakin CJ, Son GML, Inglis JT, Blouin J-S (2007) Frequency response of human vestibular reflexes characterized by stochastic stimuli. J Physiol 583:1117–1127. 10.1113/jphysiol.2007.133264 17640935 10.1113/jphysiol.2007.133264 PMC2277188

[CR8] Day BL, Séverac Cauquil A, Bartolomei L et al (1997) Human body-segment tilts induced by galvanic stimulation: A vestibularly driven balance protection mechanism. J Physiol 500:661–672. 10.1113/jphysiol.1997.sp022051 9161984 10.1113/jphysiol.1997.sp022051 PMC1159417

[CR9] Derry S, Moore RA (2014) Topical Lidocaine for neuropathic pain in adults. Cochrane Database Syst Rev CD010958. 10.1002/14651858.CD010958 10.1002/14651858.CD010958.pub2 PMC6540846 25058164

[CR11] Elliott LR, Redden ES, Pettitt RA et al (2006) Tactile guidance for land navigation. Aberdeen Proving Ground, MD

[CR12] Elliott LR, Schmeisser ET, Redden ES (2011) Development of tactile and haptic systems for U.S. infantry navigation and communication. In: Smith MJ, Salvendy G (eds) Human-Computer Interaction International Conference. Orlando, FL, pp 399–407

[CR10] Elliott LR, Mortimer B, Hartnett-Pomranky G et al (2015) Augmenting soldier situation awareness and navigation through tactile cueing. In: Yamamoto S (ed) Human-Computer Interaction International Conference. Los Angeles, CA, pp 345–353

[CR13] Fitzpatrick RC, Day BL (2004) Probing the human vestibular system with galvanic stimulation. J Appl Physiol 96:2301–2316. 10.1152/japplphysiol.00008.2004 15133017 10.1152/japplphysiol.00008.2004

[CR14] Forbes PA, Kwan A, Mitchell DE et al (2023) The neural basis for biased behavioral responses evoked by galvanic vestibular stimulation in primates. J Neurosci 43:1905–1919. 10.1523/JNEUROSCI.0987-22.2023 36732070 10.1523/JNEUROSCI.0987-22.2023 PMC10027042

[CR15] Freeman HR, Chander H, Kodithuwakku Arachchige SNK et al (2023) Postural control behavior in a virtual moving room paradigm. Biomechanics 3:539–551. 10.3390/biomechanics3040043

[CR16] Goldberg JM, Smith CE, Fernández C (1984) Relation between discharge regularity and responses to externally applied galvanic currents in vestibular nerve afferents of the squirrel monkey. J Neurophysiol 51:1236–1256 6737029 10.1152/jn.1984.51.6.1236

[CR17] Hancock PA, Lawson B, Cholewiak R et al (2015) Tactile Cuing to augment multisensory human-machine interaction. Ergon Des 23:4–9. 10.1177/1064804615572623

[CR18] Holewski JJ, Stess RM, Graf PM, Grunfeld C (1988) Aesthesiometry: quantification of cutaneous pressure sensation in diabetic peripheral neuropathy. J Rehabil Res Dev 25:1–10 3361455

[CR19] Jouira G, Alexe CI, Păun LI et al (2025) Effects of auditory environments on postural balance and cognitive performance in individuals with intellectual disabilities: A Dual-Task investigation. Appl Sci 15:486. 10.3390/app15010486

[CR20] Kwan A, Forbes PA, Mitchell DE et al (2019) Neural substrates, dynamics and thresholds of galvanic vestibular stimulation in the behaving primate. Nat Commun 10:1904. 10.1038/s41467-019-09738-1 31015434 10.1038/s41467-019-09738-1 PMC6478681

[CR23] Lee DN, Aronson E (1974) Visual proprioceptive control of standing in human infants. Percept Psychophys 15:529–532. 10.3758/BF03199297

[CR24] Lee DN, Lishman JR (1975) Visual proprioceptive control of stance. J Hum Mov Stud 1:87–95

[CR22] Lee B-C, Martin BJ, Sienko KH (2012) Directional postural responses induced by vibrotactile stimulations applied to the torso. Exp Brain Res 222:471–482. 10.1007/s00221-012-3233-2 22968737 10.1007/s00221-012-3233-2

[CR21] Lee B-C, Martin BJ, Ho A, Sienko KH (2013) Postural reorganization induced by torso cutaneous covibration. J Neurosci 33:7870–7876. 10.1523/JNEUROSCI.4715-12.2013 23637178 10.1523/JNEUROSCI.4715-12.2013 PMC6618976

[CR25] Lubetzky AV, Gospodarek M, Arie L et al (2020) Auditory input and postural control in adults: A narrative review. JAMA Otolaryngol - Head Neck Surg 146:480–487. 10.1001/jamaoto.2020.0032 32163114 10.1001/jamaoto.2020.0032

[CR26] MacDougall HG, Moore ST, Curthoys IS, Black FO (2006) Modeling postural instability with galvanic vestibular stimulation. Exp Brain Res 172:208–220. 10.1007/s00221-005-0329-y 16432695 10.1007/s00221-005-0329-y

[CR27] Manca M, Glowatzki E, Roberts DC et al (2019) Ionic direct current modulation evokes spike-rate adaptation in the vestibular periphery. Sci Rep 9:1–12. 10.1038/s41598-019-55045-6 31831760 10.1038/s41598-019-55045-6 PMC6908704

[CR28] Meyer DE, Kieras DE (1997) A computational theory of executive cognitive processes and Multiple-Task performance: part 1. Basic mechanisms. Psychol Rev 104:3–65 9009880 10.1037/0033-295x.104.1.3

[CR29] Moore ST, MacDougall HG, Peters BT et al (2006) Modeling locomotor dysfunction following spaceflight with galvanic vestibular stimulation. Exp Brain Res 174:647–659. 10.1007/s00221-006-0528-1 16763834 10.1007/s00221-006-0528-1

[CR30] Séverac Cauquil A, Martinez P, Ouaknine M, Tardy-Gervet MF (2000) Orientation of the body response to galvanic stimulation as a function of the inter-vestibular imbalance. Exp Brain Res 133:501–505. 10.1007/s002210000434 10985684 10.1007/s002210000434

[CR31] Smith KJ, Datta A, Burkhart C, Clark T (2024) Efficacy of galvanic vestibular stimulation as a display modality dissociated from self-orientation. Hum Factors 66:862–871. 10.1177/00187208221119879 35971664 10.1177/00187208221119879

[CR32] St George RJ, Fitzpatrick RC (2011) The sense of self-motion, orientation and balance explored by vestibular stimulation. J Physiol 589:807–813. 10.1113/jphysiol.2010.197665 20921198 10.1113/jphysiol.2010.197665 PMC3060360

[CR33] Steinhardt CR, Fridman GY (2021) Direct current effects on afferent and hair cell to elicit natural firing patterns. iScience 24:102205. 10.1016/j.isci.2021.102205 33748701 10.1016/j.isci.2021.102205 PMC7967006

[CR34] Stone T, Clark TK, Temple DR (2025) Noisy galvanic vestibular stimulation induces stochastic resonance in vestibular perceptual thresholds assessed efficiently using confidence reports. Exp Brain Res 243:34. 10.1007/s00221-024-06984-8 10.1007/s00221-024-06984-8 39718639

[CR35] Temple DR, Pepper S, Hogoboom BC et al (2024) Exploring GVS as a display modality: signal amplitude and Polarity, in various environments, impacts on posture, and with dual-tasking. Exp Brain Res 242:2443–2455. 10.1007/s00221-024-06908-6 39162730 10.1007/s00221-024-06908-6

[CR36] Truong DQ, Thomas C, Ira S et al (2024) Unpacking galvanic vestibular stimulation using simulations and relating current flow to reported motions: comparison across common and specialized electrode placements. PLoS ONE 19:e0309007. 10.1371/journal.pone.0309007 39186497 10.1371/journal.pone.0309007 PMC11346646

[CR37] Wehrfritz A, Namer B, Ihmsen H et al (2011) Differential effects on sensory functions and measures of epidermal nerve fiber density after application of a Lidocaine patch (5%) on healthy human skin. Eur J Pain 15:907–912. 10.1016/j.ejpain.2011.03.011 21530339 10.1016/j.ejpain.2011.03.011

[CR39] Wickens CD (1981) Processing resources in attention, dual task performance, and workload assessment. Champaign, IL

[CR40] Wickens CD (2002) Multiple resources and performance prediction. Theor Issues Ergon Sci 3:159–177. 10.1080/14639220210123806

[CR38] Wickens C (2021) Attention: theory, principles, models and applications. Int J Hum Comput Interact 37:403–417. 10.1080/10447318.2021.1874741

[CR41] Wuehr M, Peto D, Fietzek UM et al (2024) Low-intensity vestibular noise stimulation improves postural symptoms in progressive supranuclear palsy. J Neurol 271:4577–4586. 10.1007/s00415-024-12419-9 38722328 10.1007/s00415-024-12419-9 PMC11233287

